# Simulation-based training following a theoretical lecture enhances the performance of medical students in the interpretation and short-term retention of 20 cross-sectional transesophageal echocardiographic views: a prospective, randomized, controlled trial

**DOI:** 10.1186/s12909-021-02753-1

**Published:** 2021-06-09

**Authors:** Yang Zhao, Zong-Yi Yuan, Han-Ying Zhang, Xue Yang, Duo Qian, Jing-Yan Lin, Tao Zhu, Hai-bo Song

**Affiliations:** 1 Department of Anesthesiology, Affiliated Hospital of North Sichuan Medical College, Nanchong, Sichuan China; 2 Department of Anesthesiology, North Sichuan Medical College, Nanchong, Sichuan China; 3 Department of Oral and Maxillofacial, Nanchong Central Hospital, The Second Clinical Medical College of North Sichuan Medical College, Nanchong, Sichuan China; 4 Department of Anesthesiology, Pidu District People’s Hospital, 156# East Street, Pitong Town, Pidu District, Chengdu, Sichuan 611730 People’s Republic of China; 5 Department of Anesthesiology, Children’s Hospital of Chongqing Medical University, Chongqing, China; 6 Department of Anesthesiology, West China Hospital of Sichuan University, Chengdu, 610041 Sichuan People’s Republic of China

**Keywords:** Echocardiography, Transesophageal, Simulation training, Retention

## Abstract

**Background:**

Both simulation-based training and video-based training serve as educational adjuncts for learning TEE among medical students. In the present study, we hypothesized that simulation-based training would better enhance the performance of medical students in the interpretation of 20 cross-sectional views compared to video-based training.

**Methods:**

A total of 120 4th-year undergraduate medical students were enrolled in the present study. The study began with a pre-test of all the participants, followed by a 90-min theoretical lecture and a post-test. Subsequently, the participants were randomly divided into the video-based group (Group V) and simulation-based group (Group S). Next, Group V received 60 min of TEE video learning, while Group S received 60 min of TEE simulator training. After the respective training, both the groups undertook the retention-test 1 and retention-test 2, 1 week and 1 month later, respectively. The performance for each test was evaluated by five views, which were selected randomly and, respectively, from a set of 20 cross-sectional views. The primary outcome was the performance of the retention-test 1. Secondary outcomes included: (1) comparison the performances of the pre-test, post-test, and retention-test 2 between two groups; (2) comparison the performances of pre-test and post-test in the same group; (3) comparison the performances of retention-test 1, and retention-test 2 in the same group.

**Results:**

Better performances were observed in Group S in both retention-test 1 (Group V: 63.2 [52.6, 77.6] vs. Group S: 89.5 [68.4, 100.0], *P* < 0.001) and retention-test 2 (Group V: 58.0 [48.0, 72.0] vs. Group S: 74.0 [64.0, 80.0], *P* < 0.001) compared to Group V. No statistically significant differences were observed in the performances of pre-test (Group V: 8.3 [4.2, 12.5] vs. Group S: 8.3 [4.2, 12.5], *P* = 0.825) or post-test (Group V: 46.2 [38.5, 57.7] vs. Group S: 44.2 [38.5, 56.7], *P* = 0.694) between the two groups. The improvement had been observed in the post-test, compared with pre-test in the same group, respectively (Group V in post-test: 46.2 [38.5, 57.7] vs. Group V in pre-test: 8.3 [4.2, 12.5], *P* < 0.001; Group S in post-test: 44.2 [38.5, 56.7] vs. Group S in pre-test: 8.3 [4.2, 12.5], *P* < 0.001). However, the performance in retention-test 2 was significantly reduced, compared with retention-test 1 in the same group, respectively (Group V in retention-test 2: 58.0 [48.0, 72.0] vs. Group V in retention-test 1: 63.2 [52.6, 77.6] *P* = 0.005; Group S in retention-test 2: 74.0 [64.0, 80.0] vs. Group S in retention-test 1: 89.5 [68.4, 100.0], *P* < 0.001).

**Conclusions:**

Following a 90-min theoretical lecture, simulation-based training better enhanced the performance of medical students in the interpretation and short-term retention of 20 cross-sectional views compared to video-based training.

**Trial registration:**

http://www.chictr.org.cn (ChiCTR2000033519, 3/June/2020).

**Supplementary Information:**

The online version contains supplementary material available at 10.1186/s12909-021-02753-1.

## Background

Transesophageal echocardiography (TEE) is considered a valuable and useful tool for the cardiac anatomical and functional evaluation of the patients undergoing surgery, either cardiac or noncardiac [1, 2]. TEE images provide information regarding cardiac function and the effect of surgical intervention. It is essential for cardiac anesthesiologists to learn the TEE technique and properly interpret the TEE images in the perioperative period [3]. It is recommended to use 20 cross-sectional views for the basic knowledge of learning TEE [4]. The training methods for learning and interpreting TEE include lectures, simulation-based training, video-based training, actual patient training at the bedside, etc. Generally, hands-on TEE at the bedside is the “gold standard”; however, it requires repetitive exposure to practice with actual patients. Lectures may assist the trainees in understanding the basic concepts of TEE [5]. When used as an educational adjunct, video-based training has been demonstrated to improve the knowledge of interpreting echocardiographic images, although with limited retention [6]. Recently, various simulation-based training systems for TEE, cardiac catheterization, coronary angioplasty, etc., have been developed to be used by doctors and for medical education, and the application of these systems has demonstrated improvement in the related procedural capability of the healthcare providers [7]. As an educational adjunct, TEE simulation-based training has been recommended as an important approach in TEE training curriculum by practicing the echocardiography skills in a simulator [8]. However, which one among the two, video-based training and simulation-based training, is better for interpreting 20 cross-sectional views remains unclear. Since the literature recommendssimulation-based training followed by a theoretical lecture [9], we hypothesized that if followed by a theoretical lecture, simulator-based TEE training would improve the performance of novice trainees in the interpretation of 20 cross-sectional views compared to the video-based TEE training.

## Methods

### Aim

The present study aimed to compare the simulator-based TEE training with the video-based TEE training following a theoretical lecture and determined which of the two would better improve the interpretation of 20 cross-sectional views.

### Study design

The present study was designed as a single-center, randomized,, controlled, prospective clinical trial. It was conducted from April 2020 to May 2020 in the North Sichuan Medical College, Nanchong City, Sichuan Province, China, and was registered in the Chinese registry of clinical trials at http://www.chictr.org.cn (ChiCTR2000033519; 3/June/2020). The study adhered to the applicable CONSORT guidelines and was approved by the Research Ethics Committee of the Affiliated Hospital of North Sichuan Medical College (approval No. 2020/111–1).

### Population

Informed written consent was obtained from all subjects 4th-year medical students with an anesthesia major and without any previous experience in cardiac anesthesia or echocardiography were eligible for participation in the present study. Participation was allowed only after signing the consent form and agreement to adhere to the study requirements.

### Sample size

We calculated the sample size based on our pilot study, the mean ± SD of retention-test 1 performance was 59.4 ± 21.3% in the video-based training group comprising ten subjects. With a statistical power of 0.8 and a type 1 error rate of 0.05 in detecting 20% improvement as conservative, the optimum sample size was a minimum of 50 patients per group to distinctly present this difference using a two-tailed Student’s t-test. Considering a possible dropout rate of 20%, 120 patients were finally included in the present study.

### Theoretical lecture

All the participants together attended a 90-min theoretical lecture, which was taught by a tutor with experience in over 200 cases of TEE image examinations for cardiac surgeries. The lecture entailed the basic concepts of echocardiography, normal cardiac anatomy, a simple explanation of 20 cross-sectional views, and the corresponding anatomical structures.

### Randomization

After the post-test (refer to the measurement section), the participants were randomly divided (sealed envelope by SPSS with random seed 20,200,611) into the video-based group (Group V) and simulation-based group (Group S) equally .

### Intervention

The participants belonging to Group V received a 60-min video-based training (refer to the manuscript attachment, 30-min of high-definition video-watching under supervision, twice). When the video was playing, each participant had a heart model at hand to understand what was being taught. The tutor also used native language with the participants when appropriate for a better understanding. The students were not allowed to have a discussion with each other and were encouraged to ask the tutor for help if they had any confusion regarding TEE.

**Fig. 1:**
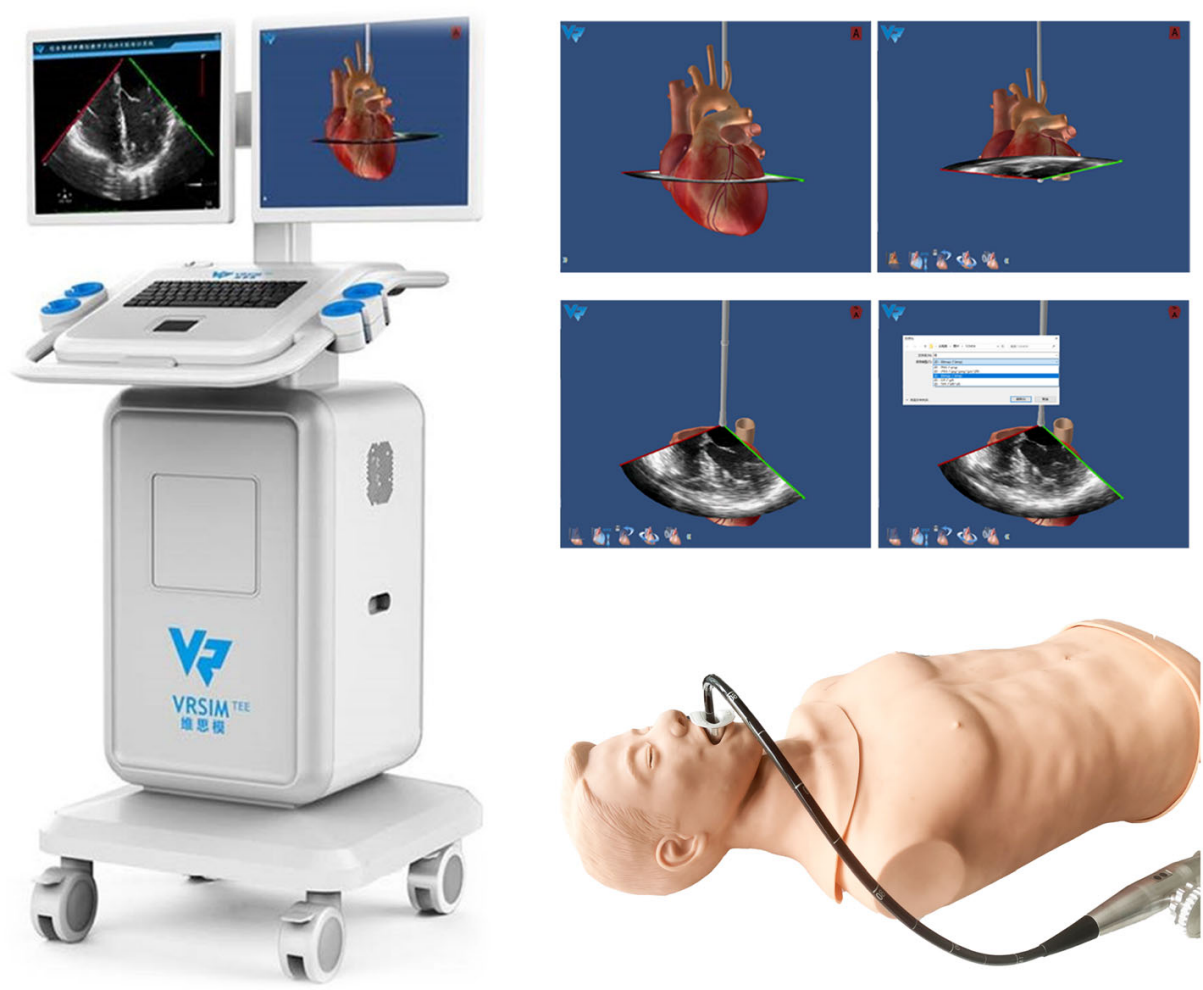
The fourth-generation of VirSim TEE training system. Note: The picture was quoted and permitted from Song H [8] who was one of the co-authors in this study. VirSim TEE training system, the fourth-generation TEE simulator, developed by Department of Anesthesiology at the West China Hospital of Sichuan University and Chengdu Branch of Chinese Academy of Sciences. The educational package of TEE simulator consists of a manikin, a TEE stationary, and a TEE simulation probe. It satisfied the needs of TEE 20 standard views for teaching and practice, cardiac clinical cases learning, 17 segments of the left ventricle learning etc.

Group S was demonstrated the 20 cross-sectional views through the simulator training system [8] (Fig. 1) for 30 min by the tutor. The training included information regarding how to move the probe to obtain each view, an explanation of the relative position of the heart, and a demonstration of how each sectional image was formed, all of which assisted the trainees in understanding every anatomical structure in the 20 cross-sectional views. The participants were allowed to undertake this training only once. Subsequently, each trainee was allowed to manipulate the TEE simulator training system alone for 30 min under the tutor’s instructions. The participants were not allowed to watch how their peers manipulat the probe or discuss with each other. Therefore, in total, each participant had 60 min of training.

The words delivered by the tutors in the 60-min training sessions were almost the same in both the groups, such as ‘this image is referred to as middle-esophagus four chambers image’, ‘when you placed the probe at the middle of the esophagus’, ‘regulate the scope into zero angle’, ‘the anatomic structure of the heart marked with the marker are the structures listed on the right part of the screen’, ‘think, which image have you got’, ‘so, the images on the screen are: left atrium, left ventricle, right atrium, right ventricle, etc.’

All participants were requested not to review the related knowledge, or discussed with peers, or learned TEE in other ways except those involved in this study during the whole period of the trial. After the training sessions, the participants were required to individually complete a checklist, which included the names and the anatomic structures of each of the 20 cross-sectional views. Only those participants who answered all the questions correctly were allowed to undergo the next trial. The participants who could not provide correct answers to all the questions were assisted by the tutor in clarifying their concepts by demonstrating and explaining the heart model for Group V or by manipulating the simulator for Group S.

### Measurements

Pre-test conducted before the training aimed at assessing the pre-existing knowledge of TEE in the participants. After the 90-min lecture, the post-test was conducted to assess the effect of the lecture. Subsequently, the participants were randomly divided into Group V and Group S (with equal participants). After completing all the training sessions, the participants were required to undertake the retention-test 1 conducted 1 week later and the retention-test 2 conducted 1 month later.

In all the tests, the participants were shown five slides in PowerPoint, each with an echocardiographic image that was depicted for a maximum of 5 min. 20 cross-sectional views included: (1) Middle esophagus ascend aortic short axis, ME Asc Aortic SAX; (2) Middle esophagus ascend aortic long axis, ME Asc Aortic LAX; (3) Middle esophagus descend aortic short axis, ME Desc Aortic SAX; (4) Middle esophagus descend aortic long axis, ME Desc Aortic LAX; (5) Upper esophagus aortic arch long axis, UE Aortic Arch LAX; (6) Upper esophagus aortic arch short axis, UE Aortic Arch SAX; (7) Middle esophagus four chamber, ME 4C; (8) Middle esophagus mitral commissural, ME MC; (9) Middle esophagus two chamber, ME 2C; (10) Middle esophagus long axis, ME LAX; (11) Middle esophagus aortic valve short axis, ME AV SAX; (12) Middle esophagus right ventricle inflow-outflow, ME RVOT;(13) Middle esophagus Bicaval, ME Bicaval; (14) Middle esophagus aortic valve long axis, ME AV LAX; (15) Transgastric basal short axis, TG Basal SAX; (16) Transgastric middle papillary short axis view, TG Mid Papillary SAX; (17) Transgastric two-chamber, TG 2C; (18) Transgastric long axis, TG LAX; (19) Transgastric right ventricle inflow, TG RV inflow; (20) Deep transgastric long axis, Deep TG LAX. Ten anatomic-structure lists included: (1) left atrium, LA (2) left ventricle, LV; (3) mitral valve, MV; (4) aortic valve, AV; (5) aorta, AO; (6) right atrium, RA; (7) right ventricle, RV; (8) tricuspid valve, TV; (9) pulmonary valve, PV; (10) pulmonary artery, PA. The participants were required to select the correct name of the image and the corresponding anatomic structure from a list of options. The number of correct answers divided by the total number of questions in each test was used to assess each test’s performance .

The pre-test included five images, which were generated using SPSS (random seed = 20,200,610): UE Aortic Arch LAX; ME MC; ME RVOT; TG RV inflow; Deep TG LAX.

The post-test included five images, which were again generated using SPSS (random seed = 20,200,611): ME Desc Aortic LAX; ME 4C; ME LAX; TG LAX; TG RV inflow.

The retention-test 1 included five images generated using SPSS (random seed = 20,200,618): ME Asc Aortic SAX; ME Desc Aortic SAX; ME 2C; ME RVOT; TG Basal SAX.

The retention-test 2 included five images generated using SPSS (random seed = 20,200,711): ME Desc Aortic SAX; ME Desc Aortic LAX; ME RVOT; ME AV LAX; TG LAX.

Details about the items involved in the tests were listed as additional materials at the end of this manuscript.

### Statistical analysis

All data were presented as median and quartiles. A 2-sample Mann-Whitney U test was used to evaluate the differences between the groups in the pre-test, post-test, retention-test 1, and retention-test 2. The paired-sample Mann-Whitney U test was used to compare the differences between pre-test and post-test, retention-test 1 and retention-test 2, respectively. The primary outcome was the performance of the retention-test 1. Secondary outcomes included: (1) comparison the performances of the pre-test, post-test, and retention-test 2 between two groups; (2) comparison the performances of pre-test and post-test in the same group; (3) comparison the performances of retention-test 1, and retention-test 2 in the same group. Statistical significance was determined using the two-tailed test at the *P*-value threshold of 0.05. All data were analyzed using the SPSS Statistics 25.0 software (Statistical Program for Social Sciences, SPSS Inc., Chicago, Illinois, USA).

## Results

**Fig. 2:**
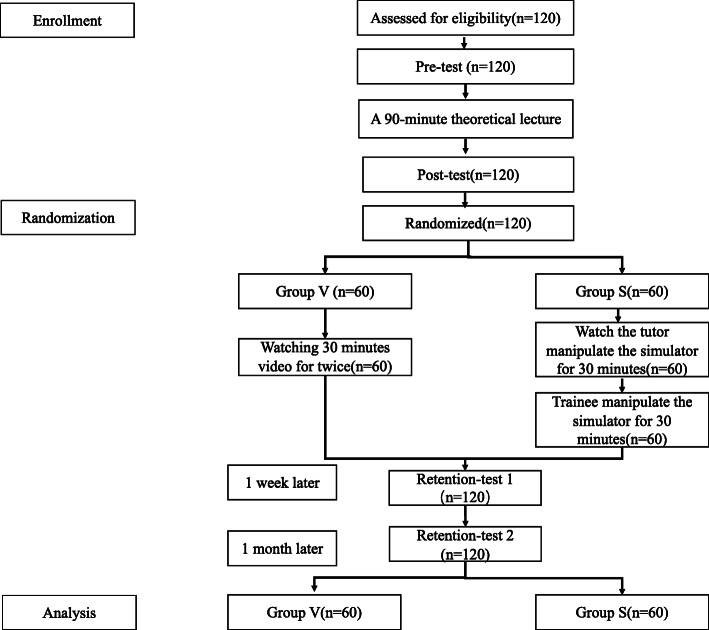
Flow chart of the TEE training sessions and tests. Note: Group V = video-based group, Group S = simulation-based group

During June 2020 and July 2020, a total of 120 4th-year undergraduate medical students with no previous experience of echocardiography from the North Sichuan Medical college were enrolled in the present study, all of whom completed the retention-test 2 conducted at the end of the trial. The flow-chart is presented in Fig. 2, and the demographic data are provided in Table 1. There was no significant difference in terms of age and sex between the two groups (Table 1).

**Table 1:** Comparison of characteristics between Group V and Group S

	Group V(*n* = 60)	Group S(*n* = 60)	*P* value
Sex (male/female)	19/41	23/37	0.444
Age (years)	22.2 ± 0.8	22.1 ± 0.9	0.755

Note: Group V = video-based group, Group S = simulation-based group

Data presented as the number for sex, mean ± standard deviation for age

**Fig. 3:**
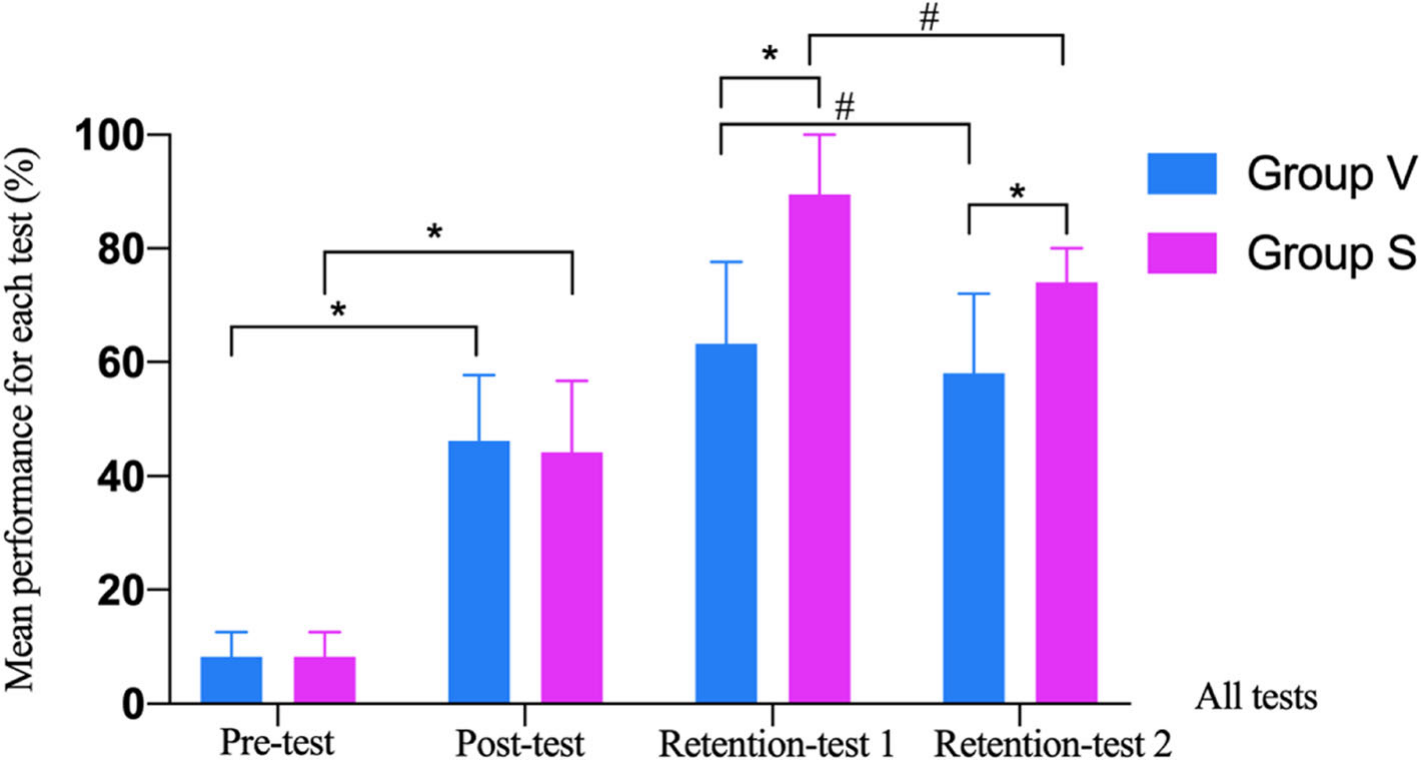
Mean performances of two groups in all the tests (%). Note:Data are expressed as median and quartiles .*means statistical significance (*P* < 0.001), #means statistical significance (*P* < 0.01)

Table 2 presents the details of the pre-test, post-test, retention-test 1, and retention-test 2. Better performance was observed in Group S in retention-test 1 (Group V: 63.2 [52.6, 77.6] vs. Group S: 89.5 [68.4, 100.0], *P* < 0.001). There were no statistically significant differences in the performances of the pre-test (Group V: 8.3 [4.2, 12.5] vs. Group S: 8.3 [4.2, 12.5], *P* = 0.825.) or post-test (Group V: 46.2 [38.5, 57.7] vs. Group S: 44.2 [38.5, 56.7], *P* = 0.694) between the two groups. Much improvement in the post-test were observed in the two groups, compared to the pre-test in the same group (paired-sample Mann-Whitney U test), which was attributed to the learning from the theoretical lecture (Group V in post-test: 46.2 [38.5, 57.7] vs. Group V in pre-test: 8.3 [4.2, 12.5], *P* < 0.001; Group S in post-test: 44.2 [38.5, 56.7] vs. Group S in pre-test: 8.3 [4.2, 12.5], *P* < 0.001). Better performance was also observed in Group S in retention-test 2 (Group V: 58.0 [48.0, 72.0], vs. Group S: 74.0 [64.0, 80.0], *P* < 0.001) compared to Group V. However, the performance in the retention-test 2 reduced in both groups, when compared to retention-test 1 in the same group (Group V in retention-test 2: 58.0 [48.0, 72.0] vs. Group V in retention-test 1: 63.2 [52.6, 77.6], *P* = 0.005; Group S in retention-test 2: 74.0 [64.0, 80.0] vs. Group S in retention-test 1: 89.5 [68.4, 100.0], *P* < 0.001. paired-sample Mann-Whitney U test). The comparisons of the two groups in all the tests are presented in Fig. 3. For more details about items and answers to all questions, which are listed at the end of the manuscript as additional materials.

**Table 2:** Comparison of mean performances (%) in each test between Group V and Group S

Views contained in each test	Group V(*n* = 60)	Group S(*n* = 60)	*P* value
Pre-test (total)	8.3 (4.2, 12.5)	8.3 (4.2, 12.5)	0.825
UE Aortic Arch LAX	0 (0, 0)	0 (0, 0)	1.000
ME MC	0 (0,75.0)	0 (0, 25.0)	0.389
ME RVOT	0 (0, 12.5)	0 (0, 12.5)	0.544
TG RV inflow	0 (0, 0)	0 (0, 0)	0.874
Deep TG LAX	0 (0, 16.7)	0 (0, 16.7)	0.082
Post-test (total)	46.2 (38.5, 57.7)^a^	44.2 (38.5, 56.7)^a^	0.694
ME Desc Aortic LAX	50.0 (0, 87.5)	50.0 (0, 50.0)	0.671
ME 4C	100.0 (100.0, 100.0)	100.0 (100.0, 100.0)	0.308
ME LAX	54.5 (25.0, 99.1)	43.8 (25.0, 100.0)	0.798
TG LAX	16.7 (0, 16.7)	0 (0, 16.7)	0.568
TG RV inflow	0 (0, 0)	0 (0, 0)	0.747
Retention test 1(total)	63.2 (52.6, 77.6)	89.5 (68.4, 100.0)	< 0.001
ME Asc Aortic SAX	16.7 (0, 100.0)	100.0 (100.0, 100.0)	< 0.001
ME Desc Aortic SAX	100.0 (100.0, 100.0)	100.0 (100.0, 100.0)	0.023
ME 2C	100.0 (100.0, 100.0)	100.0 (100.0, 100.0)	< 0.001
ME RVOT	50.0 (25.0, 100.0)	100.0 (25.0, 100.0)	0.030
TG Basal SAX	100.0 (100.0, 100.0)	100.0 (100.0, 100.0)	0.069
Retention test 2(total)	58.0 (48.0, 72.0)^b^	74.0 (64.0, 80.0)^b^	< 0.001
ME Desc Aortic SAX	100.0 (100.0, 100.0)	100.0 (100.0, 100.0)	1.000
ME Desc Aortic LAX	100.0 (100.0, 100.0)	100.0 (100.0, 100.0)	1.000
ME RVOT	56.3 (25.0, 100.0)	81.3 (25.0, 100.0)	0.298
ME AV LAX	71.4 (57.1, 85.7)	85.7 (71.4, 100.0)	< 0.001
TG LAX	0 (0, 33.3)	33.3 (33.3, 50.0)	< 0.001

Note: Data are presented as median and quartiles, analyzed by a 2-sample or paired-sample Mann-Whitney U test. ^a^Indicates the mean total performance was significantly different (*P* < 0.001, paired-sample Mann-Whitney U test) when compared with Pre-test in the same group. ^b^Indicates the mean total performance was significantly different (*P* < 0.01, paired-sample Mann-Whitney U test.) when compared with Retention test 1 in the same group. Group V = video-based training group, Group S = simulation-based training

## Discussion

The present study indicated that the students of Group S performed better in retention-test 1 and retention-test 2 for the interpretation of 20 cross-sectional views compared to the students of Group V.

The close contact of the transducer of TEE with the heart produced high-quality images in different views, allowing an easier and precise understanding of the three-dimensional heart. TEE is not only a diagnostic tool, rather a monitoring device during cardiac as well as noncardiac surgery [10]. TEE can assist the anesthesiologists in evaluating the hemodynamic status of the patients, providing sufficient information to undertake decisions related to perioperative management, and improving patient outcomes [11, 12]. It is, therefore, essential to train the anesthesiologists for TEE [13]. Recent surveys indicated that only 46% of the critical care doctors received some kind of ultrasound training, and the percentage was even lower (27.5%) among the anesthesiology residents [14, 15]. Moreover, the literature strongly recommends the introduction of ultrasound technology into medical education owing to its widespread and accessible nature of this technique and the benefit it provides to the patients, particularly its introduction in echocardiography training [16, 17].

The interpretation of 20 cross-sectional views requires the trainees to learn the normal cardiac anatomy, understand the orientation of the TEE probe, and identify each anatomic structure in the echocardiographic image. First, using the simulator training system in the present study, the tutor simultaneously demonstrated the probe spatial orientation, the corresponding three-dimensional anatomic structures, and the two-dimensional echocardiographic images. The tutor manipulated the probe on the right screen while explaining how each corresponding echocardiographic view formed vividly on the left screen. The performance in the retention-tests 1 and 2 in Group S was better than that in Group V, probably due to the hand-eye-head model used in the TEE simulator training system. This enabled the trainees to establish a correlation between the three-dimensional anatomic structures and the two-dimensional echocardiographic views quickly, thereby assisting the participants to have deeper comprehensions rather than memorization only [8]. Second, when the participants began to manipulate the probe in the simulator, the knowledge from the lecture was stimulated and organized again in the participants’ minds. The 30 min of practice on the dummy in Group S assisted the participants in putting the lecture theory and the tutor’s demonstration into practice. Finally, the tutor supervised the participants’ handling of the probe and assisted them in correcting the probe orientation and understanding the view interpretation whenever required. This correction and feedback on time assisted the participants in establishing a relationship between theory and practice. This approach followed by a lecture is based on the constructivist theory, which emphasizes learning through a cycle of doing and reflecting [18]. While theory helped the participants practice better, the practice, in turn, enhanced the understanding of the theory, resulting in a “learning cycle” [19]. This theory-practice-theory cycle could have led to better learning and retention of TEE recognition.

Video-based training has been proven to serve as an adjunct for improving the interpretation of transthoracic echocardiographic (TTE) images among novice TTE trainers [20]. Ray et al. demonstrated that, despite a lack of live skills training, the knowledge of image interpretation was improved after watching a 30-min video including the three components of didactics (transthoracic echocardiographic views, including subcostal 4-chamber view, subcostal inferior vena cava view, parasternal long-axis view, para-sternal short-axis view, apical view, and pulmonary view), virtual practice, and clinical correlation [21]. In the present study also, the performance in the retention-test 1 was significantly higher than the performance in the post-test in Group V (Group V retention-test 1: 63.2[52.6, 77.6] vs. Group V post-test: 46.2 [38.5, 57.7], *P* < 0.001), which indicated that video-based training followed a lecture improved the knowledge of interpretation of TEE images. Even though video-based learning is considered an effective teaching method with ease of access, its acceptance is limited due to a lack of actual practice [6, 22]. The video-based training conducted in our study, including image interpretation but no image acquisition practice, and it, therefore, lacked the hand-eye-head interaction and theory-practice-theory cycle, which might be the reason why the students did not have better retention of the images. Nonetheless, due to the advantages of ﻿efficiency, low cost, and ease of access, video-based training is suggested as a considerable choice when lacking a simulator, expert faculty, or sufficient practice time [23].

The performance in the retention-test 2 conducted 1 month later was significantly lower in both the groups compared to the performance in the retention-test 1, which was conducted 1 week after the training, probably because the review of the obtained knowledge was not allowed in the present trial, indicating that regular review may help retain the complicated knowledge of TEE. In addition, training on actual patients can result in serious complications to the patients, particularly those under general anesthesia who unable to speak or move during the process of inserting and manipulating the probe, such as the patients with soft-tissue infection, postoperative dysphagia, digestive tract hemorrhage, etc. [24, 25]. Using simulation at an early stage of learning for the novice trainees when the incidence of complications is the highest may effectively protect the patients from such risks [7]. TEE simulation equipment is expensive, a set of TEE training system in our study cost about 100,000 $ which can be used only in a medical training center [26], while video-based training is almost free and accessible for every practitioner. However, similar to our study, simulation-based training had been demonstrated to be an effective teaching approach, which results in better skill, knowledge acquisition, and higher training satisfaction [27]. Yet TEE simulator still has certain disadvantages. Firstly, the simulator may not satisfy the higher-level trainees as they are already proficient in the basic knowledge regarding TEE. For instance, when the trainees acquire the acceptable images, they may not know how to utilize the advanced features, such as spectral or color Doppler, using the simulator [28]. Besides, a simulator cannot replace the clinical TEE practice at the bedside of actual patients as clinical TEE enables real-time monitoring and discovers any abnormal changes in the patient, which is not possible with the simulator.

### Limitations

As with all research, the present study also had certain limitations. First, our study focused more on image interpretation rather than image acquisition. More advanced features, such as spectral or color Doppler, were not focused on in our study. Second, the long-term retention test for the interpretation of 20 cross-sectional views was not conducted. At last, we only measured the differences between simulation-based training and video-based training on novices, while it may be different for higher-level trainees. More research may be needed to explore the effect of simulation-based training on higher-level trainees in the future.

## Conclusions

Following a 90-min theoretical lecture, simulation-based training enhanced the performance of medical students in the interpretation and short-term retention of 20 cross-sectional views compared to video-based training.

## Supplementary Information

**Additional file 1: Additional material 1.** The Comparison of pre-test between Group V and Group S **Note:** Qualitative data presented as the number for the sum of trainees who responded with correct or wrong interpretation of each anatomic structure, analyzed by Chi-squared test or adjusted Chi-squared test. Continuous data presented as median and quartiles for mean total performance, analyzed by a 2-sample Mann-Whitney U test.

**Additional file 2: Additional material 2.** The Comparison of post-test between Group V and Group S **Note:** Qualitative data presented as the number for the sum of trainees who responded with correct or wrong interpretation of each anatomic structure, analyzed by Chi-squared test or adjusted Chi-squared test. Continuous data presented as median and quartiles for mean total performance, analyzed by a 2-sample Mann-Whitney U test.

**Additional file 3: Additional material 3.** The Comparison of retention-test 1 between Group V and Group S. **Note:** Qualitative data presented as the number for the sum of trainees who responded with correct or wrong interpretation of each anatomic structure, analyzed by Chi-squared test or adjusted Chi-squared test. Continuous data presented as median and quartiles for mean total performance, analyzed by a 2-sample Mann-Whitney U test.

**Additional file 4: Additional material 4.** The Comparison of retention-test 2 between Group V and Group S. **Note:** Qualitative data presented as the number for the sum of trainees who responded with correct or wrong interpretation of each anatomic structure, analyzed by Chi-squared test or adjusted Chi-squared test. Continuous data presented as median and quartiles for mean total performance, analyzed by a 2-sample Mann-Whitney U test.

## Data Availability

We declared that materials described in the manuscript, including all relevant raw data, will be freely available to any scientist wishing to use them for non-commercial purposes, without breaching participant confidentiality. Y.Z. (594624370@qq.com) would be contacted if someone wants to request the data.

## Abbreviations

TEE: Transesophageal echocardiographic
TTE: Transthoracic echocardiographic
ME Asc Aortic SAX: Middle esophagus ascend aortic short axis
ME Asc Aortic LAX: Middle esophagus ascend aortic long axis
ME Desc Aortic SAX: Middle esophagus descend aortic short axis
ME Desc Aortic LAX: Middle esophagus descend aortic long axis
UE Aortic Arch LAX: Upper esophagus aortic arch long axis
UE Aortic Arch SAX: Upper esophagus aortic arch short axis
ME 4C: Middle esophagus four chamber
ME MC: Middle esophagus mitral commissural
ME 2C: Middle esophagus two chamber
ME LAX: Middle esophagus long axis
ME AV SAX: Middle esophagus aortic valve short axis
ME RVOT: Middle esophagus right ventricle inflow-outflow
ME Bicaval: Middle esophagus Bicaval
ME AV LAX: Middle esophagus aortic valve long axis
TG Basal SAX: Transgastric basal short axis
TG Mid Papillary SAX: Transgastric middle papillary short axis view
TG 2C: Transgastric two-chamber
TG LAX: Transgastric long axis
TG RV inflow: Transgastric right ventricle inflow
Deep TG LAX: Deep transgastric long axis
LA: Left atrium
LV: Left ventricle
MV: Mitral valve
AV: Aortic valve
RA: Right atrium
RV: Right ventricle
TV: Tricuspid valve
PV: Pulmonary valve
PA: Pulmonary artery
Group S: Simulation-based group
Group V: Video-based group
