# Supplementary material for: Simulation-based training following a theoretical lecture enhances the performance of medical students in the interpretation and short-term retention of 20 cross-sectional transesophageal echocardiographic views: a prospective, randomized, controlled trial
Source: BMC Med Educ. 2021 Jun 9;21:336. doi: 10.1186/s12909-021-02753-1 (PMC8191119; doi:10.1186/s12909-021-02753-1)
Supplement: Supplementary file 1 — Additional file 1: Additional material 1. The Comparison of pre-test between Group V and Group S Note: Qualitative data presented as the number for the sum of trainees who responded with correct or wrong interpretation of each anatomic structure, analyzed by Chi-squared test or adjusted Chi-squared test. Continuous data presented as median and quartiles for mean total performance, analyzed by a 2-sample Mann-Whitney U test. [file 12909_2021_2753_MOESM1_ESM.docx]

| Answers（correct: wrong） | Group V(n=60) | Group S(n=60) | P value |
| --- | --- | --- | --- |
| UE Aortic Arch LAX | | | |
| 1 AO | 9:51 | 9:51 | 1.000 |
| 2 View name | 0:60 | 0:60 | / |
| ME MC | | | |
| 3 LA | 17:43 | 12:48 | 0.286 |
| 4 MV | 17:43 | 13:47 | 0.399 |
| 5 LV | 16:44 | 13:47 | 0.522 |
| 6 View name | 4:56 | 3:57 | 1.000 |
| ME RVOT | | | |
| 7 LA | 4:56 | 6:54 | 0.509 |
| 8 RA | 4:56 | 6:54 | 0.509 |
| 9 TV | 3:57 | 4:56 | 1.000 |
| 10 RV | 2:58 | 3:57 | 1.000 |
| 11PV | 0:60 | 0:60 | / |
| 12 PA | 0:60 | 0:60 | / |
| 13 AV | 14:46 | 12:48 | 0.658 |
| 14 View name | 2:58 | 2:58 | 1.000 |
| TG RV inflow | | | |
| 15 RA | 4:56 | 4:56 | 1.000 |
| 16TV | 4:56 | 4:56 | 1.000 |
| 17 RV | 7:53 | 7:53 | 1 |
| 18 View name | 0:60 | 0:60 | / |
| Deep TG LAX. | | | |
| 19 LA | 3:57 | 6:54 | 0.488 |
| 20 MV | 3:57 | 6:54 | 0.488 |
| 21 LV | 7:53 | 8:52 | 0.783 |
| 22AV | 4:56 | 6:54 | 0.509 |
| 23AO | 4:56 | 6:54 | 0.509 |
| 24View name | 0:60 | 0:60 | / |
|  |  |  |  |
| Mean total performance（%） | 0 (0, 16.7) | 0 (0, 16.7) | 0.082 |
