# Supplementary material for: Simulation-based training following a theoretical lecture enhances the performance of medical students in the interpretation and short-term retention of 20 cross-sectional transesophageal echocardiographic views: a prospective, randomized, controlled trial
Source: BMC Med Educ. 2021 Jun 9;21:336. doi: 10.1186/s12909-021-02753-1 (PMC8191119; doi:10.1186/s12909-021-02753-1)
Supplement: Supplementary file 2 — Additional file 2: Additional material 2. The Comparison of post-test between Group V and Group S Note: Qualitative data presented as the number for the sum of trainees who responded with correct or wrong interpretation of each anatomic structure, analyzed by Chi-squared test or adjusted Chi-squared test. Continuous data presented as median and quartiles for mean total performance, analyzed by a 2-sample Mann-Whitney U test. [file 12909_2021_2753_MOESM2_ESM.docx]

| Answers（correct: wrong） | Group V(n=60) | Group S(n=60) | P value |
| --- | --- | --- | --- |
| ME Desc Aortic LAX | | | |
| 1AO | 42:18 | 37:23 | 0.336 |
| 2View name | 13:47 | 15:45 | 0.666 |
| ME 4C | | | |
| 3RA | 58:2 | 55:5 | 0.436 |
| 4TV | 58:2 | 56:4 | 0.675 |
| 5RV | 58:2 | 57:3 | 1.000 |
| 6LA | 55:5 | 52:8 | 0.378 |
| 7MV | 58:2 | 59:1 | 1.000 |
| 8LV | 59:1 | 56:4 | 0.361 |
| 9View name | 58:2 | 57:3 | 1.000 |
| ME LAX | | | |
| 10LA | 43:17 | 35:25 | 0.126 |
| 11MV | 33:27 | 26:34 | 0.201 |
| 12LV | 26:34 | 29:31 | 0.583 |
| 13AV | 32:28 | 37:23 | 0.356 |
| 14AO | 32:28 | 37:23 | 0.356 |
| 15RV | 26:34 | 29:31 | 0.583 |
| 16View name | 27:33 | 21:39 | 0.264 |
| TG LAX | | | |
| 17LA | 12:48 | 11:49 | 0.817 |
| 18MV | 5:55 | 5:55 | 1 |
| 19LV | 12:48 | 18:42 | 0.206 |
| 20AV | 0:60 | 0:60 | / |
| 21AO | 0:60 | 0:60 | / |
| 22View name | 11:49 | 10:50 | 0.810 |
| TG RV inflow | | | |
| 23RA | 4:56 | 5:55 | 1.000 |
| 24TV | 4:56 | 5:55 | 1.000 |
| 25RV | 4:56 | 5:55 | 1.000 |
| 26 View name | 4:56 | 4:56 | 1.000 |
|  |  |  |  |
| Mean overall performance（%） | 46.2 (38.5, 57.7) | 44.2 (38.5,56.7) | 0.694 |
