# Supplementary material for: Simulation-based training following a theoretical lecture enhances the performance of medical students in the interpretation and short-term retention of 20 cross-sectional transesophageal echocardiographic views: a prospective, randomized, controlled trial
Source: BMC Med Educ. 2021 Jun 9;21:336. doi: 10.1186/s12909-021-02753-1 (PMC8191119; doi:10.1186/s12909-021-02753-1)
Supplement: Supplementary file 3 — Additional file 3: Additional material 3. The Comparison of retention-test 1 between Group V and Group S. Note: Qualitative data presented as the number for the sum of trainees who responded with correct or wrong interpretation of each anatomic structure, analyzed by Chi-squared test or adjusted Chi-squared test. Continuous data presented as median and quartiles for mean total performance, analyzed by a 2-sample Mann-Whitney U test. [file 12909_2021_2753_MOESM3_ESM.docx]

| Answers (correct: wrong） | Group V(n=60) | Group S(n=60) | P value |
| --- | --- | --- | --- |
| ME Asc Aortic SAX |  |  |  |
| 1 PA | 26：34 | 50：10 | <0.0001 |
| 2 AO | 27：33 | 50：10 | <0.0001 |
| 3 View name | 21：39 | 48：12 | <0.0001 |
| ME Desc Aortic SAX | | | |
| 4 AO | 55：5 | 60：0 | 0.068 |
| 5 View name | 55：5 | 60：0 | 0.068 |
| ME 2C | | | |
| 6 LA | 53：7 | 60：0 | 0.019 |
| 7 MV | 53：7 | 60：0 | 0.019 |
| 8 LV | 51：9 | 60：0 | 0.006 |
| 9 View name | 27：33 | 42：28 | 0.006 |
| ME RVOT | | | |
| 10 LA | 29：31 | 43：17 | 0.009 |
| 11 RA | 30：30 | 43：17 | 0.015 |
| 12 TV | 29：31 | 42：18 | 0.016 |
| 13 RV | 36：24 | 42：18 | 0.251 |
| 14 PV | 28：32 | 39：21 | 0.043 |
| 15 PA | 28：32 | 39：21 | 0.043 |
| 16 AV | 45：15 | 52：8 | 0.104 |
| 17 View name | 48：12 | 52：8 | 0.327 |
| TG Basal SAX | | | |
| 18 MV | 51：9 | 57：3 | 0.068 |
| 19View name | 51：9 | 57：3 | 0.068 |
|  |  |  |  |
| Mean overall performance（%） | 63.2 (52.6, 77.6) | 89.5 (68.4,100.0) | <0.001 |
