# Supplementary material for: Simulation-based training following a theoretical lecture enhances the performance of medical students in the interpretation and short-term retention of 20 cross-sectional transesophageal echocardiographic views: a prospective, randomized, controlled trial
Source: BMC Med Educ. 2021 Jun 9;21:336. doi: 10.1186/s12909-021-02753-1 (PMC8191119; doi:10.1186/s12909-021-02753-1)
Supplement: Supplementary file 4 — Additional file 4: Additional material 4. The Comparison of retention-test 2 between Group V and Group S. Note: Qualitative data presented as the number for the sum of trainees who responded with correct or wrong interpretation of each anatomic structure, analyzed by Chi-squared test or adjusted Chi-squared test. Continuous data presented as median and quartiles for mean total performance, analyzed by a 2-sample Mann-Whitney U test. [file 12909_2021_2753_MOESM4_ESM.docx]

| Answers（correct: wrong） | Group V(n=60) | Group S(n=60) | P value |
| --- | --- | --- | --- |
| ME Desc Aortic SAX |  |  |  |
| 1 AO | 60:0 | 60:0 | / |
| 2 View name | 60:0 | 60:0 | / |
|  |  |  |  |
| ME Desc Aortic LAX | | | |
| 3 AO | 60:0 | 60:0 | / |
| 4 View name | 60:0 | 60:0 | / |
| ME RVOT | | | |
| 5 LA | 26:34 | 33:27 | 0.201 |
| 6 RA | 31:29 | 38:22 | 0.196 |
| 7 TV | 30:30 | 37:23 | 0.198 |
| 8 RV | 36:24 | 37:23 | 0.852 |
| 9 PV | 27:33 | 30:30 | 0.583 |
| 10 PA | 27:33 | 32:28 | 0.361 |
| 11 AV | 51:9 | 55:5 | 0.255 |
| 12 View name | 52:8 | 53:7 | 0.783 |
| ME AV LAX | | | |
| 13 LA | 43:17 | 58:2 | <0.001 |
| 14 MV | 45:15 | 57:3 | 0.002 |
| 15 LV | 45:15 | 60:0 | <0.001 |
| 16 AV | 59:1 | 60:0 | 0.315 |
| 17 AO | 59:1 | 60:0 | 1.000 |
| 18 RV | 28:32 | 36:24 | 0.143 |
| 19 View name | 8:52 | 34:26 | <0.001 |
| TG LAX | | | |
| 20 LA | 7:53 | 14:46 | 0.093 |
| 21 MV | 5:55 | 11:49 | 0.107 |
| 22 LV | 27:33 | 52:8 | <0.001 |
| 23 AV | 5:55 | 12:48 | 0.067 |
| 24 AO | 5:55 | 11:49 | 0.107 |
| 25 View name | 22:38 | 53:7 | <0.001 |
|  |  |  |  |
| Mean overall performance（%） | 58.0 (48.0, 72.0) | 74.0 (64.0, 80.0) | <0.001 |
